# Multimodal Imaging in Diagnosing Multiple Evanescent White Dot Syndrome following Human Papillomavirus Vaccine Immunization

**DOI:** 10.1155/2024/9600771

**Published:** 2024-01-19

**Authors:** Jing Yu, Yuying Ji, Yunkao Zeng, Huihui Li, Hailan Liao, Feng Wen

**Affiliations:** ^1^Ophthalmology Department of The Second Affiliated Hospital, School of Medicine, The Chinese University of Hong Kong, Shenzhen, China; ^2^Longgang District People's Hospital of Shenzhen, Shenzhen 518172, China; ^3^State Key Laboratory of Ophthalmology, Zhongshan Ophthalmic Center, Sun Yat-sen University, Guangdong Provincial Key Laboratory of Ophthalmology and Visual Science, Guangzhou 510060, China; ^4^Department of Ophthalmology, The Second Affiliated Hospital of Guangzhou Medical University, No. 250 Changgang East Road, Haizhu District, Guangzhou City 510260, China

## Abstract

*Purpose*. This study presents a case of multiple evanescent white dot syndrome (MEWDS) following the administration of the second dose of a human papillomavirus vaccine (HPV). We conducted a review of the literature on vaccine-associated MEWDS. *Observations*. A 23-year-old Chinese female reported central scotomata in the left eye persisting for 3 weeks. Upon further inquiry, she had received the second dose of the human papillomavirus vaccine (Gardasil-9) three days before the onset of symptoms. A diagnosis of MEWDS was established based on clinical and multimodal imaging (MMI) data. Symptoms resolved after twelve weeks of oral prednisone treatment. *Conclusion and Importance*. This case highlights a typical case of MEWDS closely associated with HPV vaccination, demonstrating a favorable prognosis with MMI. Given the self-limiting nature of MEWDS, there is a risk of clinical misdiagnosis or oversight. While further studies are warranted to establish a definitive link between the HPV vaccine and MEWDS, this case suggests a potential connection. Healthcare practitioners should remain vigilant regarding possible ocular side effects associated with immunizations.

## 1. Introduction

While uncommon, there have been accumulating reports of vaccine-associated uveitis as a local adverse effect of vaccinations [1]. Cases of multiple evanescent white dot syndrome (MEWDS) have been documented following various vaccinations [2], with a notable increase in reports associated with the introduction of COVID-19 vaccines. This study presents a case of MEWDS following the administration of the second dose of a human papillomavirus vaccine. Additionally, we conducted a comprehensive review of all reported cases of vaccine-associated MEWDS and compared the findings.

## 2. Case Report

**Figure 1:**
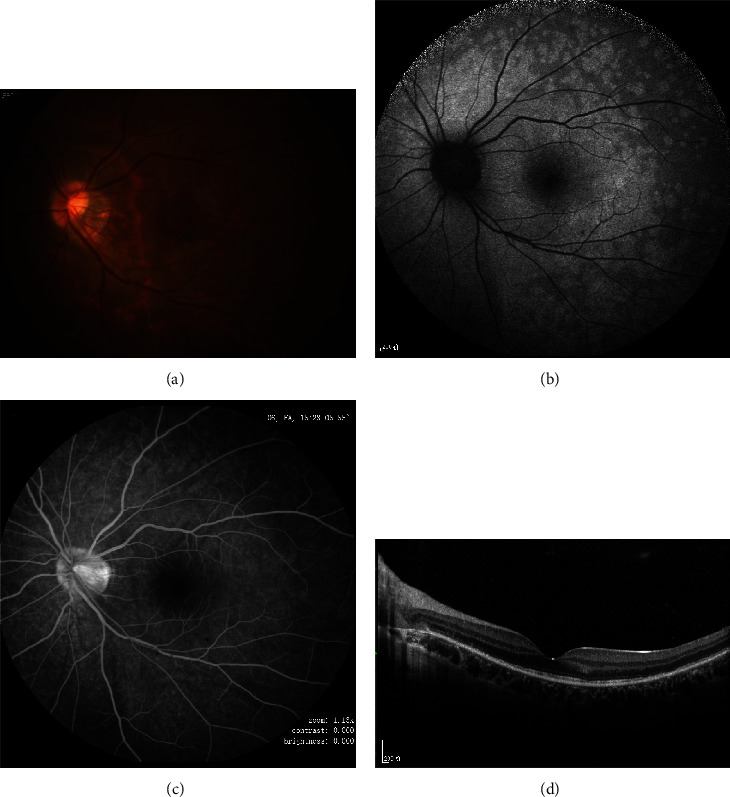
The initial presentation: (a) color fundus photo, (b) fundus autofluorescence, (c) fluorescence angiography, and (d) optical coherence tomography.

We report the case of a 23-year-old Chinese female who presented with central scotomata in her left eye (OS) persisting for three weeks. Initially diagnosed with xerophthalmia at a local hospital, her symptoms did not improve with sodium hyaluronate eye drop treatment. Subsequently, she sought evaluation at the Zhongshan Ophthalmic Center of Sun Yat-sen University. Upon ophthalmic examination, her OD (right eye) exhibited a best-corrected visual acuity (BCVA) of 15/100, while her OS (left eye) demonstrated a BCVA of 20/20 with refractive errors of +2.50DS/-0.25DC155 and -5.00DS/-1.25DC170, respectively. Intraocular pressure measured 11 mmHg in both eyes, and extraocular motility was normal. Pupils were evenly rounded and responsive to light. Slit-lamp examination revealed normal anterior segments and vitreous. A tessellated fundus was also observed (OS) (Figure 1(a)). Fundus autofluorescence (FAF) exhibited numerous mixed hyperfluorescent patches, dispersed hyperfluorescent lesions, and small hyperfluorescent circles, all centered around the optic disc and posterior pole (OS) (Figure 1(b)). Optical coherence tomography (OCT) revealed diffuse damage in the ellipsoid zone (EZ) near the macula and punctate hyperreflective lesions of varying sizes in the outer retina (Figure 1(d)). Fluorescein angiography (FA) depicted early punctate hyperfluorescence in a wreath-like pattern with late staining (Figure 1(c)). The diagnosis of MEWDS was established based on the clinical and multimodal imaging (MMI) data. Upon further inquiry, the patient reported receiving the second dose of a human papillomavirus (HPV) vaccine (Gardasil-9) three days before the onset of symptoms. The patient denied experiencing flu-like symptoms and had a history of amblyopia in the right eye, with no remarkable past medical or family histories.

**Figure 2:**
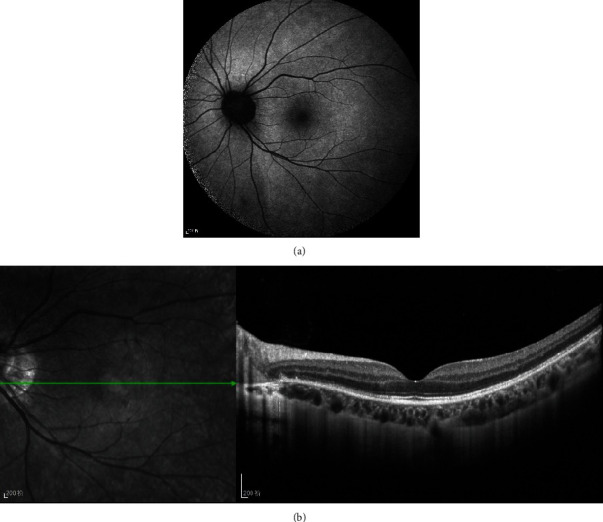
Four weeks later, (a) FAF has less hyperfluorescent spots. (b) OCT findings demonstrated improved EZ and less punctate hyperreflective lesions.

**Figure 3:**
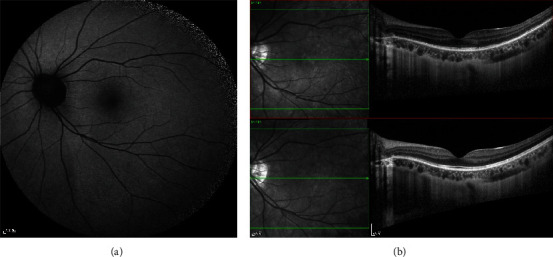
Twelve weeks later, compared with the initial presentation, (a) the hyperfluorescent spots in FAF disappeared completely. (b) OCT demonstrated EZ recovery.

A trial of prednisone 20 mg per day was administered for 1 week and then tapered gradually. Four weeks later, she said the central scotomata were getting better. In FAF, there were less hyperfluorescent spots (Figure 2(a)). OCT findings demonstrated improved EZ and less punctate hyperreflective lesions in the left (Figure 2(b)). Her BCVA was 0.15 (15/100) on the right and 1.0(20/20) on the left at that time. Twelve weeks after the initial presentation, she said the central scotomata had already disappeared. In addition, the hyperfluorescent spots in FAF disappeared completely (Figure 3(a)). OCT demonstrated EZ recovery in OS (Figure 3(b)).

## 3. Discussion

MEWDS, initially described by Jampol et al. in 1984 [3], is a relatively uncommon condition characterized by the unilateral presence of numerous pale yellow lesions in the outer retina and retinal pigment epithelium, often accompanied by foveal orange granularity. Typically affecting young individuals, particularly women [4], up to 50% of MEWDS cases may exhibit flu-like symptoms preceding ocular involvement. The condition has been associated with influenza and various immunizations, and in most instances, spontaneous resolution occurs within 8 to 10 weeks [5].

In this case, it is highly conceivable that the patient was initially misdiagnosed, leading to an absence of recorded fundus data during the early stages. When she presented at Zhongshan Ophthalmic Center, no specific fundus changes were noted. However, subsequent clinical and multimodal imaging findings supported the diagnosis of MEWDS. The patient, a previously healthy young woman, developed a central scotoma in her myopic eye (OS) three days after receiving an HPV vaccination. The clinical characteristics and MMI results aligned with MEWDS. HPV vaccines, given as preventative measures, are an affordable technique that can lower the incidence of cervical cancer. Gardasil-9, licensed by the FDA in 2014, provides defense against HPV6, 11, 16, 18, 31, 33, 45, and 58 (Merck & Co., Kenilworth, NJ, USA). It has been reported that Gardasil-9 has the potential to prevent almost 90% of cervical cancers due to the five new types it covers, which could include HPV strains linked to an additional 20% of occurrences of cervical cancer [6]. The HPV vaccine is administered in three doses over the course of a series [7].

Two studies have discussed the incidence of MEWDS following HPV vaccination [8, 9]. In one study, a 16-year-old girl developed throat soreness, headache, and photopsia (OS) two weeks after her second HPV shot (Cervarix®, Glaxo Smith Kline). Two months later, the white dots had mostly vanished, but the patient's visual field had deteriorated, and she reported losing her peripheral vision gradually over a two-year period. The authors explored the possibility of a coexisting disease entity, such as acute zonal occult outer retinopathy. In the other study, a previously healthy 17-year-old girl with myopia reported seeing dark shimmering spots (OS) for three days. Only her left eye was affected. She had received her first meningococcal and HPV vaccinations one month before the onset of vision loss, which resolved without treatment in eight weeks.

Ng et al. reported a median duration of 14 days (range: 1-30 days) between immunization and MEWDS [2]. Despite most MEWDS cases having an infectious trigger before ocular symptoms emerge, our patient received the second dose of the HPV vaccine merely three days before symptom onset, suggesting the probable role of ocular inflammation induced by vaccination in this MEWDS case. In contrast to the previous two cases, we present the first instance of MEWDS after the nine-valent HPV vaccination, with multimodal imaging findings. The nine-valent vaccine, containing more target proteins and virus-like particles, covering additional HPV subtypes, and incorporating more adjuvants, potentially increases the likelihood of adverse events in patients [6]. Our patient's vaccination was more temporally relevant to MEWDS onset. In contrast to a previous case with a good prognosis who received both meningococcal and HPV vaccines, our patient, receiving only the HPV vaccine, also experienced a good prognosis. Another case that received only the HPV vaccine, however, showed a poor prognosis, and the possibility of a different concurrent disease entity should have been considered by the authors.

Over the years, several MEWDS cases have recovered following vaccination with various medications. The COVID-19 outbreak has led to the development of different vaccines, and some cases of MEWDS have been reported after COVID-19 vaccination. We have summarized and compared all the cases in Table 1.

There have been twenty-four published MEWDS cases after different vaccines, including rabies [10], human papillomavirus [8, 9], hepatitis A [11], hepatitis B [12], meningococcal [9], yellow fever [13], influenza [14], and COVID-19 [15–25]. Patients with postvaccination MEWDS were mostly healthy (79.2%), youthful to middle-aged (mean 35.9 years; median 33.5 years; range 15–71 years) women (66.7%). 91.7% of patients received the inactive vaccine. Symptoms manifested on average 11.1 days (median: 8.5; range: 1-30) after immunization. Mean presenting the Snellen visual acuity was of 20/34 (median: 20/30; range: 20/400-20/16). A spontaneous return to baseline Snellen visual acuity was seen in seventeen cases (70.8%) of postvaccine MEWDS after an average of 5.9 weeks (median: 6 weeks; range: 2-12 weeks). Ten cases (41.6%) of MEWDS developed after receiving the second or the third dose of vaccination.

According to Bolletta et al. [23], 58.8% of ocular problems caused by COVID-19 immunization were discovered after the second dosage. Renisi et al. [26] found that while local reactogenicity was similar for both vaccine dosages, systemic reactogenicity was more prevalent and severe after the second dose. The US Centers for Disease Control and Prevention (CDC) cautioned that the second dose of the BNT162b2 vaccine is linked to an increased risk of adverse effects throughout the body. In one study [27], roughly 60% of recipients reported experiencing symptoms such as fever, headache, myalgia, and general malaise following the second dose. Uveitis following the second dose of the COVID-19 vaccine may occur, as observed in this case, indicating a possible connection between immunization and MEWDS. Different vaccines may have common pathogenesis, and several articles on molecular mimicry [28], hypersensitivity reactions [29], and autoimmunity induced by adjuvants (ASIA) [30] have been written to illustrate this phenomenon. Nevertheless, establishing a definitive link between the vaccine and MEWDS proved challenging, and it remains possible that the eye irritation following immunization was a coincidence.

The potential pathogenesis of the patient's condition may have been as follows: after the initial vaccination dose, the body became sensitized and produced a small amount of antibodies. With the second dose, the antigen-antibody reaction intensified. Additionally, myopia may have contributed to a thinner retinal pigment epithelium, potentially allowing antibodies to reach the photoreceptor and cause MEWDS. The patient's immune function may also have played a role in MEWDS development.

## 4. Conclusions

Overall, the relationship between the HPV vaccine and MEWDS warrants further study. Vaccines may cause MEWDS by inducing an autoimmune response. Prophylactic vaccinations against concomitant disorders have proven to be the most cost-effective techniques for lowering disease incidence. Since the advent of the smallpox vaccine, humans have benefitted greatly from various vaccinations. Despite their rarity and minimal likelihood of adverse effects, the advantages of vaccinations exceed the risks. Indeed, doctors should be aware of any potential ocular side effects from such immunizations.

## Figures and Tables

**Table 1:** Summary of all cases of MEWDS following vaccination.

Study	Vaccine name	Age	Gender	Time from vaccination (days)	Vaccine type	Comorbidities	Presenting VA in affected eye (Snellen)	Vision at last visit in the affected eye	Intervention	Time until resolution (weeks)
Baglivo et al.[10]	Hepatitis B booster 3rd dose	23	F	1	Recombinant vaccines	None	20/200	Undisclosed-“recovery of vision”	None	12

Fine et al.[11]	Hepatitis A	33	M	13	Inactivated vaccines	None	20/25-2	20/20	None	6

Stangos et al. [12]	Hepatitis A and yellow fever	50	F	10	Inactivated vaccines	None	20/40	20/20	None	6

Cohen [9]	Human papilloma virus and meningococca	17	F	30	Recombinant vaccines	None	20/200	20/20	None	8

Goyal et al. [13]	Influenza	53	M	10	Undisclosed	Hepatitis B and C infection, polysubstance abuse	20/25-2	20/20	None	4

Ogino et al. [8]	Human papilloma virus (Cervarix®, Glaxo Smith Kline) 2nd dose	16	F	14	Recombinant vaccines	None	20/16	20/16	None initially, betamethasone and antihistamine later for peripheral vascular leakage and associated visual field constriction	Retinal lesions resolved at 2 months; worsening peripheral vision loss for 2 years

Abou-Samra et al. [14]	Influenza	27	F	14	Undisclosed	Stevens-Johnson syndrome, Wolff-Parkinson-White, vesicourethral reflux, mild chronic kidneydisease, endometriosis, fibroadenomas, depression with anxiety	20/25-2	Undisclosed	None	8

Yang et al. [15]	Rabies 3rd dose	33	F	14	Embryonated-egg vaccine	None	20/20	20/20	Retrobulbar triamcinolone acetonide 40 mg	Partially resolved at 8 weeks

Ng et al. [2]	Influenza (Flucelvax Quadrivalent® Seqirus)	34	M	14	Inactivated vaccines	None	20/20+2	20/16	None	4

Rabinovitch et al. [16]	COVID-19 2nd dose	39	M	5	mRNA vaccines	None	20/32	20/20	None	8
		28	F	30	mRNA vaccines	None	20/32	20/20	None	8

Xu and Shen [17]	COVID-19 1st dose	49	F	2	Inactivated vaccines	MEWDS	20/100	20/20	Oral prednisone	8

Inagawa et al. [18]	COVID-19 1st dose	30	F	6	mRNA vaccines	None	20/20	20/20	Topical corticosteroid	8

Yasuda et al. [19]	COVID-19 2nd dose	67	F	1	mRNA vaccines	None	20/100	20/25	None	2

Lin and Hsieh [20]	COVID-19 1st dose	36	F	2	Recombinant vaccines	None	20/25	20/20	None	4

Tomishige et al. [21]	COVID-19 1st dose	38	F	7	Inactivated vaccines	None	20/400	20/20	Oral prednisone	4

Smith et al. [22]	COVID-19 2nd dose	15	M	14	mRNA vaccines	None	20/100	20/20	Oral prednisone	2
		21	F	21	mRNA vaccines	None	20/60	20/20	Oral prednisone	2

Bolletta et al. [23]	COVID-19 2nd dose	53	M	28	mRNA vaccines	None	20/25	20/20	None	Undisclosed time but complete resolution
	COVID-19 1st dose	18	F	4	mRNA vaccines	None	20/66	20/20	None	Undisclosed time but complete resolution
	COVID-19 1st dose	48	M	7	mRNA vaccines	None	20/400	20/20	None	Undisclosed time but complete resolution

Alhabshan and Scales [24]	COVID-19 3rd dose	71	F	3	mRNA vaccines	Retinal tear was treated with laser barricade; hysterectomy, hypercholesterolemia	20/30	Undisclosed	None	6

Gargouri et al.[25]	COVID-19 1st dose	23	F	10	mRNA vaccines	SARS-CoV-2 infection	20/20	20/20	None	6
		40	M	7	mRNA vaccines	None	20/25	Undisclosed	None	6

*N* = 24	41.7% developed after receiving the second or the third dose vaccination	Mean:35.9 Median: 33.5 Range:15–71	66.7% female	Mean:11.1 Median: 8.5 Range: 1–30	91.7% of vaccines are inactive	79.2% none	Mean: 20/34 Median: 20/30 Range: 20/400–20/16	Mean: 20/20 Median: 20/20 Range: 20/25–20/16	70.8% none	Mean: 5.9 Median: 6 Range: 2–12

## Data Availability

Data are available on request.
